# Open-chest Pulsed Electric Field Ablation of Cardiac Ganglionated Plexi in Acute Canine Models

**DOI:** 10.19102/icrm.2022.130704

**Published:** 2022-07-15

**Authors:** Martin van Zyl, Mariam Khabsa, Jason A. Tri, Thomas P. Ladas, Omar Z. Yasin, Adetola O. Ladejobi, John Reilly, Barry O’Brien, Kenneth Coffey, Samuel J. Asirvatham

**Affiliations:** ^1^Department of Cardiovascular Medicine, Mayo Clinic, Rochester, MN, USA; ^2^AtriAN Medical Ltd., Galway, Ireland; ^3^Departments of Pediatric Cardiology, Laboratory Medicine, and Pathology, Mayo Clinic, Rochester, MN, USA

**Keywords:** Ganglionated plexi, electroporation, pulsed electric fields, atrial fibrillation, cardiac denervation

## Abstract

This study aimed to evaluate the safety and acute effect on markers of cardiac autonomic tone following pulsed electric fields (PEFs) delivered to epicardial ganglionated plexi (GP) during a cardiac surgical procedure. Ablation of GP as a treatment for atrial fibrillation (AF) has shown promise, but thermal ablation energy sources are limited by the risk of inadvertent collateral tissue injury. In acute canine experiments, median sternotomy was performed to facilitate the identification of 5 epicardial GP regions using an anatomy-guided approach. Each site was targeted with saline-irrigated PEF (1000 V, 100 μs, 10 electrocardiogram [ECG]-synchronized pulse sequences). Atrial effective refractory period (AERP) and local electrogram (EGM) amplitude were measured before and after each treatment. Histology was performed on samples from treatment-adjacent structures. In 5 animals, 30 (n = 2) and 60 (n = 3) pulses were successfully delivered to each of the 5 target sites. There was no difference in local atrial EGM amplitude before and after PEF application at each site (1.83 ± 0.41 vs. 1.92 ± 0.53 mV, *P* = .72). The mean AERP increased from 97 ± 15 ms at baseline to 115 ± 7 ms following treatment at all sites (18.6% increase; 95% confidence interval, 1.9–35.2; *P* = .037). There were no sustained ventricular arrhythmias or acute evidence of ischemia following delivery. Histology showed complete preservation of adjacent atrial myocardium, phrenic nerves, pericardium, and esophagus. Use of PEF to target regions rich in cardiac GP in open-chest canine experiments was feasible and effective at acutely altering markers of cardiac autonomic tone.

## Introduction

Atrial fibrillation (AF) is a common occurrence following cardiac surgery. Approximately 1/3 of patients will develop early postoperative AF in the first week after surgery, even in the absence of a prior AF diagnosis.^1^ In the immediate postoperative setting, AF is linked to increased rates of renal dysfunction, encephalopathy, and infection and prolonged hospital and intensive care unit stays.^1^ Chronically, postoperative AF has been independently associated with a risk of AF later in life as well as increased cardiovascular mortality.^2^ Whether long-term risks can be modified by a strategy focusing on the prevention of postoperative AF remains a topic of debate.

Contemporary AF management strategies focus on either pharmacological or ablative manipulation of atrial myocardial conduction and refractoriness. Surgical ablation using cryotherapy, radiofrequency ablation (RFA), or cut-and-sew maze techniques have been widely described and are generally considered to be effective. Yet, the added surgical risk and operative time impede the use of these techniques in patients without prior atrial dysrhythmias. Furthermore, surgical ablation relies on myocardial tissue destruction to achieve lines of conduction block and are increasingly being recognized as having potential deleterious long-term hemodynamic and arrhythmic implications.^3–5^

The cardiac autonomic nervous system has been strongly implicated in the initiation and perpetuation of AF. The inputs of this nervous system coalesce in a complex network of interconnecting nerves to form the ganglionated plexi (GPs). These GPs are concentrated epicardially in anatomic regions, which have been described previously through systematic in vivo stimulation and microscopic histology.^6–12^ Based on preclinical and clinical experimentation, GP stimulation and heightened parasympathetic drive result in heterogeneous atrial action potential shortening, decreased conduction velocities, reduced refractory periods, and calcium-induced triggered activity.^13–18^ This recipe of conditions can promote induction and maintenance of AF even in healthy hearts.

Partial cardiac autonomic denervation by means of GP ablation has become an attractive potential treatment for AF as an adjunct or alternative to traditional myocardial ablative approaches. This has been attempted through both surgical and minimally invasive means. Several trials have relied on the use of RFA energy and have yielded conflicting results in efficacy for the treatment of AF.^18–24^ Thermal energy risks inadvertent collateral injury to surrounding structures, including the atrial myocardium, esophagus, phrenic nerves, and coronary arteries. Atrial myocardial scarring could explain the observation of increased macro–re-entrant atrial flutters following RFA of GPs in a trial.^21^ Furthermore, the high impedance encountered as a result of the epicardial fat in which GPs reside may make these structures relatively resistant to RFA.^25^

Pulsed electric fields (PEFs) have recently gained substantial traction in cardiac applications. With PEFs, ablation is achieved through induction of apoptosis via the non-thermal process of irreversible electroporation. Preclinical studies have demonstrated focused ablation of targeted tissues using specific energy parameters while sparing non-target tissues such as the pulmonary veins, the esophagus, coronary arteries, and the phrenic nerves.^26–29^

Our group previously evaluated the acute safety and efficacy of anatomy-guided GP ablation using PEFs in canines via percutaneous epicardial access.^30,31^ We hypothesized that PEFs delivered in an open-chest setting would similarly result in targeted ablation of GPs while avoiding collateral injury to the atrial myocardium and other surrounding structures.

## Methods

The study protocol was approved in advance by the Mayo Clinic Institutional Animal Care and Use Committee under compliance with the Guide for the Care and Use of Laboratory Animals.

### Animal preparation and access

Acute experimental studies were performed in 5 male mongrel canines (body weight, 25–35 kg). The animals were fasted overnight prior to the procedure. Anesthesia was induced with intramuscular tiletamine–zolazepam (5 mg/kg) followed by intravenous diazepam (0.5 mg/kg) and ketamine (10 mg/kg). The animals were intubated, supported with mechanical ventilation, and maintained on inhaled isoflurane (2%–5%) with continuous intravenous fentanyl (2 μg/kg/h) for additional analgesia. Adequate sedation and analgesia were ensured by monitoring for changes in heart rate and blood pressure from baseline.

The skin was prepared by shaving the anterior chest, surface electrocardiogram (ECG) electrode sites, and a site over the upper lumbar spine to allow placement of an adhesive dispersive return patch (Valleylab PolyHesive E7507; Covidien, Minneapolis, MN, USA). Using the percutaneous Seldinger technique, standard 9-French (Fr) intravascular sheaths were placed in the right femoral and right jugular veins, and an 18-gauge arterial pressure-monitoring line was placed in the left femoral artery. A median sternotomy was performed using a combination of sharp and blunt dissection to expose the sternum, the sternum was transected with trauma shears, and hemostasis was achieved with the use of electrocautery and bone wax applied to the cut bone edges. A sternal retractor was used to expose the mediastinum. The anterior visceral pericardium was incised and, using anchored sutures circumferentially, the heart was exposed. The heart was kept covered with warm saline-moistened towels when direct visualization was not required. The esophageal temperature was measured at the level of the left atrium and maintained at 36.5°C–37.5°C with the use of forced air warming. The pericardial recesses and thoracic cavity were kept free of excessive fluid accumulation by intermittent handheld suction.

### Establishing the atrial effective refractory period

The atrial effective refractory period (AERP) was used as a surrogate for atrial autonomic tone using similar methods to prior preclinical studies.^30–34^ Three separate pacing sites distant to PEF energy delivery were used to assess AERP, as follows: endocardial high right atrium (HRA), distal coronary sinus (CS), and left atrial appendage (LAA). A quadripolar standard curve, 8-Fr, 8-mm tip catheter (Blazer II XP; Boston Scientific Corp., Marlborough, MA, USA) was placed in the HRA position and a 7-Fr deflectable duodecapolar catheter (Orbiter ST; Boston Scientific Corp.) was inserted into the distal CS. A 2-0 temporary cardiac pacing wire (Ethicon, Somerville, NJ, USA) was sutured into the base of the LAA and a second wire was placed into the subcutaneous tissue at the inferior margin of the sternotomy incision. Electrograms (EGMs) were recorded and analyzed using the Prucka CardioLab EP System (General Electric Healthcare, Chicago, IL, USA). Unipolar local EGMs were recorded from the ablation catheter electrode to Wilson’s Central Terminal as a reference. The atrial potential amplitude was measured from peak to peak in millivolts (mV). Bipolar pacing was performed from the distal electrodes of the HRA catheter and the most distal electrode pair of the CS catheter, which would allow consistent atrial capture. Epicardial unipolar pacing was performed from the LAA pacing wire using the subcutaneous wire as a return. AERP was established at baseline in each of the 3 pacing sites using an 8-cycle pacing train at a cycle length of 500 ms with a single extra-stimulus and a coupling interval shortened progressively at 10-ms intervals. When the sinus heart rate remained at >120 bpm consistently, the pacing train cycle length was decreased to 400 ms. AERP was defined as the longest coupling interval for which the extra-stimulus failed to capture the atrial myocardium.

### Targeted anatomic locations

**Figure 1:**
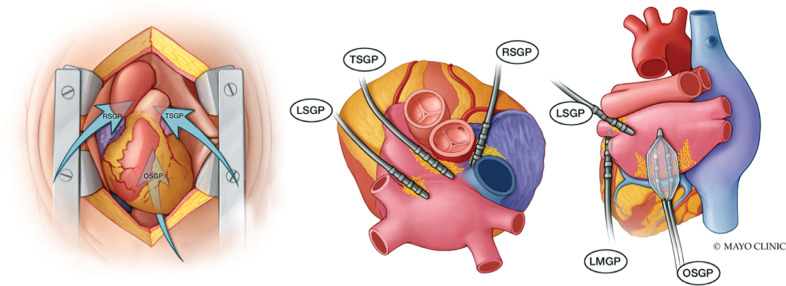
Target anatomical regions known to be rich in clinically important ganglionated plexi. Ganglionated plexi (*yellow dots*) with roles important to initiation or maintenance of AF were targeted with pulsed electric field delivery through an open-chest anatomic approach. *Abbreviations:* LMGP, ligament of Marshall ganglionated plexi; LSGP, left superior ganglionated plexi; OSGP, oblique sinus ganglionated plexi; RSGP, right superior ganglionated plexi; TSGP, transverse sinus ganglionated plexi.

Based on anatomic descriptions from prior studies, including prior publications from our group, 5 epicardial regions known to contain GPs with important roles in AF were targeted **(Figure 1)**.^6–9,11,18,30^ Each site was treated sequentially in the order outlined below. The nomenclature for these GP-containing regions varies widely in the literature, and the anatomic descriptions and nomenclature for the purposes of this study were defined as follows:

1. Oblique sinus GP (OSGP)—found on the posterior wall of the left atrium adjacent to the pulmonary veins within the oblique sinus of the pericardium.
2. Right superior GP (RSGP)—located on the anterosuperior surface of the right atrium, rightward of the aortic root and leftward of the superior vena cava.
3. Transverse sinus GP (TSGP)—contained within the transverse pericardial sinus with the roots of the great arteries anteriorly and pulmonary veins posteriorly. Separated anatomically from the RSGP by the dome of the right atrial roof at the right-sided entrance of the transverse pericardial sinus.
4. Left superior GP (LSGP)—found at the junction of the left superior pulmonary vein and the superior left atrium.
5. Ligament of Marshall GP (LMGP)—located between the anterior aspect of the left inferior pulmonary vein and the posterior LAA in the region of the ligament of Marshall.

### Pulsed electric field delivery

**Figure 2:**
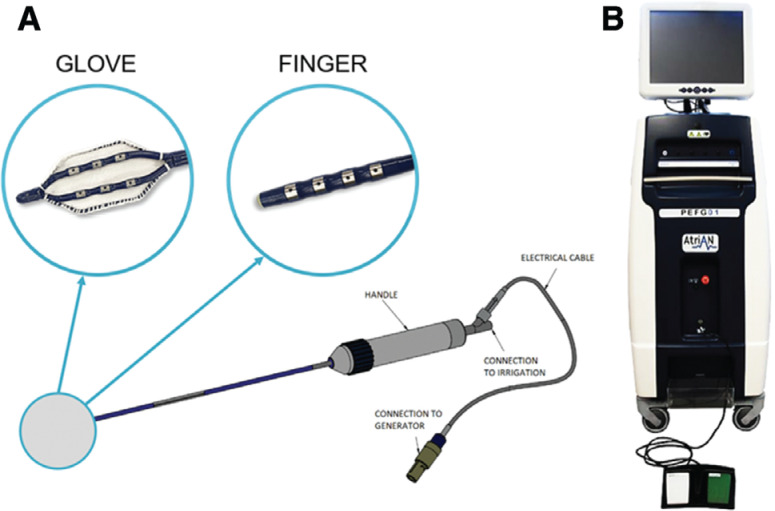
Catheters and pulsed electric field generator. **A:** AtriAN catheters used for pulsed electric field with 2 different tip elements designed to reach target treatment sites. **B:** AtriAN PEFG01 pulsed electric field generator.

Two purpose-designed catheters (AtriAN Medical Ltd., Galway, Ireland) were used for PEF energy delivery **(Figure 2A)**. Unidirectional deflectable shafts allowed navigation and electrode contact onto intended treatment sites. Two catheter tip element types were used depending on the treated site. The Glove catheter (used only in the OSGP region) had 6 irrigated electrodes connected in parallel, which were exposed on the treatment surface and covered on the non-treatment surface by a polyester woven mesh. The Finger catheter had 4 irrigated electrodes with shielding on the non-treatment surface and was used in all other regions.

Continuous irrigation with 0.9% saline was performed through the catheter ports at 2 mL/min using a peristaltic pump (CoolFlow; Biosense Webster, Diamond Bar, CA, USA). The AtriAN PEFG01 generator (AtriAN Medical Ltd.) **(Figure 2B)** was used to deliver smooth waveform direct current energy PEF in 1000-V, 100-μs, 10-pulse sequences—parameters previously shown to be effective at achieving percutaneous AERP extension.^31^ Each pulse was synchronized to the R-wave of the surface ECG using a cardiac trigger monitor (model 7600; Ivy Biomedical Systems, Branford, CT, USA). The number of total pulses delivered to each target site was varied (30 in animals #1 and #2 and 60 in animals #3, #4, and #5, respectively). Energy was delivered in a unipolar fashion between the catheter electrodes and the dispersive return patch. Vecuronium bromide (0.05 mg/kg) was given intravenously 5 min prior to energy delivery and as needed every 30 min as a paralytic in order to diminish skeletal muscle contractions. Since vecuronium has been shown to possess weak ganglion-blocking activity at very high doses (vagal/neuromuscular blockade ED50 ratio of 96), AERP was assessed before and after paralytic administration at the start of each study.^35^

EGMs were re-analyzed following energy delivery. AERP was re-established following energy delivery to each target site using the same method outlined above in all 3 pacing sites within 5 min of treatment and again at 5 min following completion of all sites. The animals were euthanized by induction of ventricular fibrillation at the end of each acute experimental procedure.

### Histologic analysis

The heart and surrounding structures were harvested during necropsy and grossly examined. The atrial myocardium beneath each of the 5 treatment sites was further sectioned and fixed in neutral buffered 10% formalin. Adjacent segments of the phrenic nerve, pericardium, and esophagus were also sectioned and fixed. Histology was processed with hematoxylin and eosin as well as Masson’s trichrome staining to assess for acute structural disruption. Staining for immunohistochemical markers of apoptosis (such as caspase-3) was not performed given the short survival of animals following sternotomy and low expression < 6 h following PEF in prior studies.^36^ Slides were evaluated blindly by a single board-certified pathologist.

### Statistical analysis

Continuous variables were summarized using mean ± standard deviation (SD) values and categorical variables were summarized with numbers and percentages. Outcome values of interest before and after treatment were compared using a paired *t* test. A 2-sided *P* ≤ .05 result was considered to be statistically significant. All analyses were conducted using GraphPad Prism 9.0.1 (GraphPad Software, San Diego, CA, USA).

## Results

### Pulsed electric field delivery

The experimental protocol was successful in all 5 animals over 248 ± 20 min of total procedure time. Less than 100 mL of blood loss occurred during access in all animals except #5, where inadvertent injury to a branch of the left internal mammary artery resulted in 400 mL of blood loss controlled by tying the vessel proximally. PEF energy was successfully delivered to all 5 target sites in all animals over 46 ± 12 min, with 30 pulses delivered per site in animals #1 and #2 and 60 pulses delivered per site in animals #3, #4, and #5. The current response recorded on the AtriAN generator system was typically 10 ± 2 amps with each pulse. There were no sustained ventricular or atrial arrhythmias induced by energy delivery. Prominent but transient (<5 min) blood pressure increases (systolic > 20 mmHg or mean > 10 mmHg) and heart rate increases (>20 bpm) were seen after delivery in 19 out of 25 and 21 out of 25 sites, respectively. In animal #4, systolic blood pressure responses exceeded 200 mmHg before returning to baseline at 5 min. Raw vital sign and AERP data were recorded for each animal by treatment site before and after PEF energy delivery **(Table 1)**.

### Electrogram analysis

**Figure 3:**
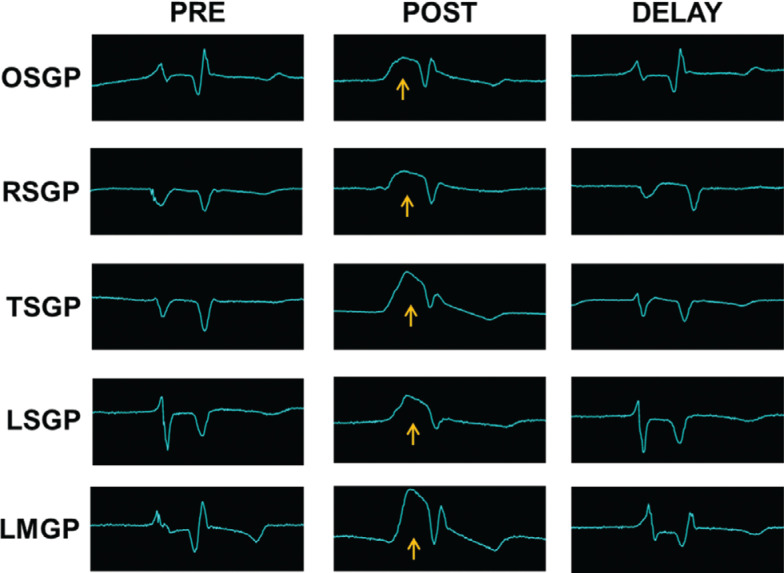
Transient atrial current of injury. Electrograms recorded from the energy delivery catheters at baseline, immediately following delivery, and after a 10-min delay in a representative animal (#3). Note the transient atrial injury current (yellow arrow) between atrial and ventricular potentials on the local unipolar electrogram followed by recovery of signal amplitude and morphology to approximate baseline. *Abbreviations:* LMGP, ligament of Marshall ganglionated plexi; LSGP, left superior ganglionated plexi; OSGP, oblique sinus ganglionated plexi; RSGP, right superior ganglionated plexi; TSGP, transverse sinus ganglionated plexi.

On local unipolar EGMs, an atrial current of injury (defined as a transient elevation of a previously isoelectric segment between local atrial and ventricular EGMs) was recorded over 21 out of 25 ablation sites. The current of injury recovered to baseline within 10 min in all 21 sites where this was noted **(Figure 3)**. At 10 min, there was no difference in local atrial EGM amplitude before (1.83 ± 0.41 mV) and after PEF energy delivery (1.92 ± 0.53 mV; *P* = 0.72) averaged across each site. There was no surface ECG evidence of ST elevation or depression following delivery.

### Atrial effective refractory periods

**Figure 4:**
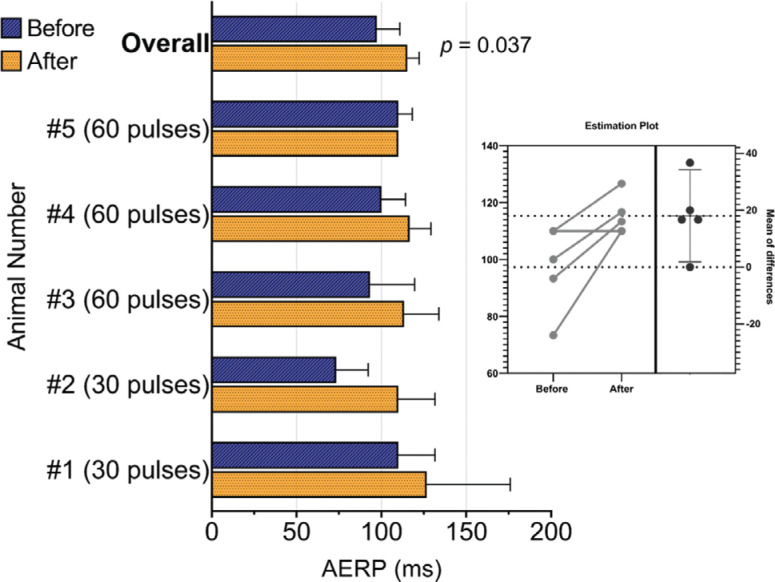
Pre- and post-treatment AERP analysis. AERP stratified per animal and combined overall before and after pulsed electric field delivery to all target sites with *t* test estimation plot. *Abbreviation:* AERP, atrial effective refractory period.

The average AERP increased by 18 ± 12 ms from 97 ± 15 ms at baseline to 115 ± 7 ms following treatment at all sites (**Figure 4**; 18.6% increase; 95% confidence interval, 1.9–35.2; *P* = .037). Stratified by pacing site, AERP acutely increased by 10 ms (12%) at the HRA, 12 ms (12%) at the CS, and 32 ms (25%) at the LAA. In animals where 30 pulses were delivered per site (n = 2), the AERP was increased by 27 ms (29%) as opposed to 12 ms (12%) in those where 60 pulses were delivered (n = 3). The sample size was not large enough to draw conclusions regarding differences in pacing site and the number of pulses delivered. There was no effect on AERP before and after vecuronium administration in any of the 5 animals.

In animal #2, recurrent AF occurred with pacing at shorter coupling intervals while attempting to establish AERP. AF terminated spontaneously. For this animal, in 4 out of 15 pacing sites, AERP was recorded as the longest coupling interval which did not result in atrial arrhythmia. In animal #3, recurrent atrial flutter occurred during pacing which did not spontaneously terminate, and 10-J synchronized cardioversion through epicardial paddles resulted in restoration of sinus rhythm on 5 separate occasions. During the sixth cardioversion attempt before the last delivery site (LMGP), the animal went into ventricular fibrillation. After 4 min of cardiac massage, 1 mg of intravenous epinephrine, and 3 defibrillation attempts, sinus rhythm was restored, and the protocol was completed.

### Histologic analysis

**Figure 5:**
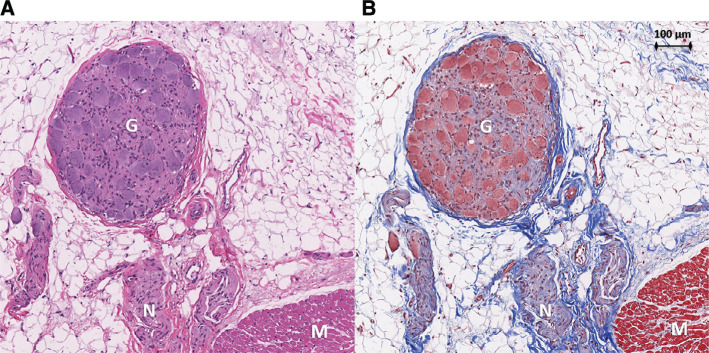
Treatment site histology. Histology of an atrial section at the treated oblique sinus ganglionated plexus region in animal #4 prepared with hematoxylin and eosin (**A**) as well as Masson’s trichome (**B**) stains. Nerve fibers (*N*) and a ganglion (*G*) are shown within epicardial fat adjacent to atrial myocardium (*M*). Tissue architecture is preserved without cellular disarray or nuclear disintegration.

In total, 35 samples were sent for histological evaluation, including 18 sections of atrial myocardium directly below the treated sites in all 5 locations, 6 adjacent phrenic nerve segments, 3 adjacent esophageal segments, and 3 sections of pericardium. A blinded review of the histology showed completely preserved atrial myocardium with intact nuclei and no evidence of acute cellular disarray or nuclear disintegration. Scattered acute ecchymoses were seen consistent with mechanical trauma from tissue manipulation during the procedure in 4 of 18 (22%) of the atrial myocardial preparations. Coronary arteries, when present, were widely patent without thrombus. Nervous tissue and ganglia were identified in the epicardial fat of the atrial myocardium in 17 of 18 (94%) and 8 of 18 (44%) sections, respectively. Nervous tissue and ganglia did not show acute structural disruption **(Figure 5)**. Esophageal, phrenic nerve, and pericardial tissue architectures were normal in appearance.

## Discussion

This study demonstrates the results of PEF energy delivery to GPs using an anatomic approach in a series of canine median sternotomy surgical procedures. Autonomic tone as measured by AERP was affected acutely without any change in local atrial EGM amplitude to suggest acute myocardial injury. This was accomplished with the use of a novel PEF generator and catheter combination.

### Pulsed electric fields for ablation of ganglionated plexi

As opposed to thermal energy sources for GP ablation, PEF energy has the potential benefit of preferential tissue effects at a given set of energy delivery parameters. Germane to the current study, PEF energy could ablate epicardial GPs while sparing sensitive surrounding structures. Similar to prior publications, PEF energy delivery was not associated with clinical or histologic signs of collateral injury to non-target adjacent structures such as the phrenic nerves, esophagus, and coronary arteries.^27–29^ In addition, the high impedance encountered as a result of the epicardial fat in which GPs reside makes these structures relatively resistant to RFA, which may in part explain some disappointing results with prior trials using this modality.^25^ Cells within fatty tissue have been shown to be amenable to penetration and ablation by PEF.^37^

Unlike conventional myocardial ablation methods for AF, such as the maze procedure, GP ablation does not aim to directly cause atrial myocardial injury or scarring. Yet, thermal ablation energy sources are inherently limited through the mechanism of inducing coagulative necrosis in directly adjacent tissue. The inadvertent result is atrial scarring and fibrosis in these target regions, which can contribute to pulmonary vein stenosis, stiff left atrial syndrome, or scar-related pro-arrhythmia.^3–5,22^ Acute EGM amplitude reduction of 50%–60% has been associated with permanent atrial myocardial ablation.^26,38,39^ In this study, local atrial amplitude remained unchanged following PEF energy delivery. This suggests that effects to the myocardium with the delivered parameters are likely to be reversible with a reduced potential for permanent myocardial ablation as shown in prior chronic experiments.^30,31^ Furthermore, no evidence of acute tissue disruption was seen on histology with the caveat that observation of myocardial irreversible electroporation by histology could be limited by the acute nature of this study. Although threshold electric field strength for inducing irreversible electroporation does not differ significantly for myocardial or neuronal cells in vitro, it is conceivable that a proximity effect may play a role in the ablation of epicardial GPs directly adjacent to the electrodes while sparing myocardium underlying layers of epicardial connective and adipose tissue in vivo.^40^

### Acute effects on autonomic tone

In addition to other proposed mechanisms, ablation of GPs has been postulated to prevent the induction of AF through withdrawal of the vagal influence on myocardium and the direct prolongation of the atrial refractory period.^33^ As such, assessing AERP confers an indirect measure of cardiac parasympathetic innervation while offering direct insight into the propensity for AF. In this study, a significant AERP extension of nearly 20% occurred following treatment. Similar AERP prolongation in a chronic canine study has been associated with evidence of durable histological GP ablation.^31^ The threshold AERP extension for effective long-term prevention of AF has not been established. Another study utilizing RFA for GP ablation has shown that, despite an acute increase in AERP, a marked reduction occurs at 4 weeks, suggesting potential for cardiac reinnervation.^41^ Whether this observation also holds true for PEF remains to be shown.

Prominent blood pressure and heart rate responses were frequently but inconsistently seen following energy delivery. The significance of this is unclear and may have represented a manifestation of discomfort during treatment. However, animals were deeply sedated, and such responses were not seen following other potentially painful stimuli such as skin incision and sternotomy. One hypothesis is that pulsed electric field delivery in these locations with rich autonomic innervation may have transiently stimulated sympathetic afferents, resulting in a pronounced but temporary systemic sympathetic response.

### Transient current of injury

Despite an absence of sustained change in the local atrial EGM amplitude, a prominent but transient elevation of a previously isoelectric interval between local atrial and ventricular potentials was recorded on local unipolar EGMs after PEF energy delivery in most sites **(Figure 3)**. The appearance is similar to the current of injury described following active fixation pacing/defibrillation lead implantation in which delayed current is thought to flow from mechanically injured to uninjured cells following depolarization.^42^ Prior studies of cardiac PEF energy delivery have not reported this finding from local EGM analysis. Transient ST elevation on surface ECG leads has been reported in a small human study after PEF energy delivery to the pulmonary veins.^43^ The authors hypothesized that this occurred through a mechanism of transmyocardial membrane potential differences as a result of enhanced depolarization near the cathode and hyperpolarization near the anode and not through ischemia or damage to the myocardium. ST elevation following direct current cardioversion is also a well-reported occurrence which has not been associated with long-term myocardial systolic dysfunction.^44,45^ The significance of this transient finding and whether the underlying mechanism is a result of reversible cellular electroporation remain unclear.

### Limitations

All acute surrogate markers of cardiac autonomic tone, including AERP, have some limitations. Extended open-chest surgical times, blood loss associated with sternotomy, cardioversion, and ventricular fibrillation are known physiologic stressors which would be expected to enhance sympathetic tone and endogenous catecholamine release, in turn reducing AERP. Animal #5 experienced more blood loss than the others, resulting in transient hemodynamic instability requiring fluid resuscitation. This animal had no change in AERP before and after treatment. Atrial extra-stimulus testing to establish AERP can induce atrial arrhythmias even in healthy hearts. Animal #2 had cardioversion-related ventricular fibrillation, and exogenous epinephrine was administered during resuscitation prior to the last pacing site. These factors would be expected to decrease AERP and should have shifted the results toward the null hypothesis.

The acute design limited the ability to assess durable effects on AERP as well as histological changes secondary to apoptosis as a result of irreversible electroporation. Rather, histological data from this study excluded evidence of thermal injury, barotrauma, or mechanical disruption during surgical manipulation. In addition, these were healthy canines, and it is unclear whether the results of this study would apply to human patients with cardiac disease requiring cardiac surgery and a predisposition to AF. Finally, there was no sham control group to isolate and compare the effects of the surgical procedure alone without delivery of PEF.

## Conclusion

The use of PEF to target regions rich in cardiac GPs in acute open-chest canine experiments was feasible and effective at altering markers of cardiac autonomic tone. Reduced risk of off-target tissue injury favors PEF as an alternative to thermal energy modalities for GP ablation. This carries potential as a novel treatment for AF during cardiac surgical procedures.

## Figures and Tables

**Table 1:** Detailed Baseline and Post-treatment Data

Animal #	Site	Target Energy Dose	No. of Pulses Delivered	Peak Heart Rate (bpm)	Blood Pressure S/D/M (mmHg)	AERP HRA (ms)	AERP CS (ms)	AERP LAA (ms)
**1**	Baseline	N/A	N/A	113	79/51/60	80	120	130
	OSGP	1000 V 100 μs	10 × 3	121	152/110/124	60	120	130
	RSGP	1000 V 100 μs	10 × 3	142	182/141/155	50	120	170
	TSGP	1000 V 100 μs	10 × 3	111	163/112/129	50	120	170
	LSGP	1000 V 100 μs	10 × 3	127	174/137/149	60	120	170
	LMGP	1000 V 100 μs	10 × 3	121	127/93/104	70	120	190
**2**	Baseline	N/A	N/A	104	75/45/55	60	100*	60
	OSGP	1000 V 100 μs	10 × 3	108	102/68/79	60	130	90*
	RSGP	1000 V 100 μs	10 × 3	162	111/74/86	90*	130	120*
	TSGP	1000 V 100 μs	10 × 3	164	110/77/88	50	90	110
	LSGP	1000 V 100 μs	10 × 3	141	82/58/66	60	110	100
	LMGP	1000 V 100 μs	10 × 3	138	125/86/99	80	130	120
**3**	Baseline	N/A	N/A	117	81/67/72	70	80	130
	OSGP	1000 V 100 μs	10 × 6	111	240/172/195	100	80	140
	RSGP	1000 V 100 μs	10 × 6	128	238/168/191	110	110	130
	TSGP	1000 V 100 μs	10 × 6	123	195/148/164	130	120	130
	LSGP	1000 V 100 μs	10 × 6	133	182/142/155	100	100	160
	LMGP	1000 V 100 μs	10 × 6	122	162/126/138	110	90	140
**4**	Baseline	N/A	N/A	94	106/63/77	90	120	90
	OSGP	1000 V 100 μs	10 × 6	107	156/120/132	120	150	110
	RSGP	1000 V 100 μs	10 × 6	116	145/111/122	130	130	90
	TSGP	1000 V 100 μs	10 × 6	114	139/113/122	120	110	100
	LSGP	1000 V 100 μs	10 × 6	98	129/120/123	120	120	150
	LMGP	1000 V 100 μs	10 × 6	84	124/98/106	100	130	120
**5**	Baseline	N/A	N/A	152	80/55/63	120	100	110
	OSGP	1000 V 100 μs	10 × 6	137	81/56/64	140	130	150
	RSGP	1000 V 100 μs	10 × 6	128	95/54/68	170	100	120
	TSGP	1000 V 100 μs	10 × 6	142	110/72/85	150	100	130
	LSGP	1000 V 100 μs	10 × 6	144	101/62/75	130	100	140
	LMGP	1000 V 100 μs	10 × 6	149	96/65/75	110	110	110

*Abbreviations:* AERP, atrial effective refractory period; CS, coronary sinus; HRA, high right atrium; LAA, left atrial appendage; LMGP, ligament of Marshall ganglionated plexi; LSGP, left superior ganglionated plexi; OSGP, oblique sinus ganglionated plexi; RSGP, right superior ganglionated plexi; S/D/M, systolic/diastolic/mean; TSGP, transverse sinus ganglionated plexi. Vital signs and atrial effective refractory period response are stratified by each experimental animal and each energy delivery site. *Denotes values that were likely higher than the actual AERP due to pacing-induced arrhythmia interfering with interpretation.
